# Supplementary material for: Emergence and control of photonic band structure in stacked OLED microcavities
Source: Nat Commun. 2021 Oct 20;12:6111. doi: 10.1038/s41467-021-26440-3 (PMC8528838; doi:10.1038/s41467-021-26440-3)
Supplement: Supplementary file 4 — Supplementary Data 1 [file 41467_2021_26440_MOESM4_ESM.zip › OLED Simulation v2-1/OLED Simulation/Materials Data/Materials Database/rii-database-2019-02-11.zip!/database/info/main/Fe3O4.html]

# Iron(II,III) oxide, Fe_3_O_4_

## Other names

- Ferrous ferric oxide
- Ferroso ferric oxide
- Black iron oxide
- Rust
- Iron(II) diiron(III) oxide
- Iron oxide
- Triiron tetraoxide

## Minerals

- Magnetite
- Lodestone (naturally magnetized magnetite)

## External links

- Iron(II,III) oxide - Wikipedia
- Triiron tetraoxide - WebElements
- Magnetite - Wikipedia
- Lodestone - Wikipedia
- Rust - Wikipedia
